# Evaluation of EPID-Based Transmission and DLG Correction Methods for Dosimetric Verification in HyperArc Single-Isocenter Multiple Target Radiosurgery

**DOI:** 10.3390/diagnostics16162628

**Published:** 2026-08-19

**Authors:** Se An Oh, Sung Yeop Kim, Jae Won Park, Ji Woon Yea, Jaehyeon Park, Yoon Young Jo

**Affiliations:** 1 Department of Radiation Oncology, Yeungnam University Medical Center, Daegu 42415, Republic of Korea; tgs01178@ynu.ac.kr (S.Y.K.); kapicap@yu.ac.kr (J.W.P.); yjw1160@ynu.ac.kr (J.W.Y.); drjhyeon@ynu.ac.kr (J.P.); cantabile801@naver.com (Y.Y.J.); 2 Department of Radiation Oncology, College of Medicine, Yeungnam University, Daegu 42415, Republic of Korea

**Keywords:** HyperArc, single-isocenter multiple targets (SIMT), portal dosimetry, electronic portal imaging device (EPID), dosimetric leaf gap (DLG), radiosurgery

## Abstract

**Background/Objectives**: Accurate verification of single-isocenter multiple-target (SIMT) stereotactic radiosurgery is challenging owing to the complexity of multi-lesion delivery and the sensitivity of stereotactic dose gradients. We aimed to evaluate the efficacy of electronic portal imaging device (EPID)-based multileaf collimator (MLC) transmission and dosimetric leaf gap (DLG) correction for improving portal-dose prediction agreement in SIMT stereotactic radiosurgery (SRS) and to propose an exploratory target count-based institutional action level. **Methods**: This retrospective analysis included 112 consecutive patients treated with HyperArc™-based SRS (1–13 targets). Treatment plans were calculated using the Acuros XB algorithm for 6 MV flattening filter-free (FFF) beams. Two sets of MLC parameters were compared for portal-dose image prediction (PDIP): (1) standard parameters measured using an ion chamber; (2) EPID-derived corrected parameters. Portal-dose accuracy was evaluated using gamma index analysis with a 95% pass rate threshold. **Results**: The uncorrected method showed a strong negative correlation between target number and gamma passing rates, with complex plans (≥9 targets) dropping as low as 70%. EPID-based correction substantially improved dose agreement, yielding consistent passing rates above 98%, regardless of target number. Although uncorrected parameters remained within tolerance for plans with one to two targets, accuracy declined markedly starting at three targets. **Conclusions**: Our findings indicate that the EPID-based correction of MLC transmission and DLG mitigated cumulative modeling discrepancies in complex SIMT SRS. Thus, we propose the exploratory institutional observation of three targets, beyond which EPID-based correction may be considered to achieve optimal portal-dose prediction agreement in HyperArc-based stereotactic radiosurgery.

## 1. Introduction

The single-isocenter multiple targets (SIMT) technique has been widely adopted for treating multiple brain metastases because it improves treatment efficiency and shortens delivery time [1,2,3,4,5,6]. Previously, managing multiple intracranial lesions required multiple planning isocenters, which substantially increased treatment time and heightened the risk of intrafractional patient motion. The transition to SIMT has transformed stereotactic radiosurgery (SRS) by enabling simultaneous delivery of highly conformal doses to disparate lesions within a single coordinate system, thereby maximizing patient comfort and clinical throughput [7]. By consolidating what was once a succession of individual treatment setups into a unified session, clinics can maintain rigorous throughput while minimizing mechanical wear from frequent repositioning. However, this consolidation demands precise spatial accuracy of the multileaf collimator (MLC), as the geometric penalty for even microscopic misalignment scales proportionally with the distance from the single coordinate origin, presenting unique physical challenges not encountered in traditional configurations.

Building on these advances, HyperArc™ (Varian Medical Systems, Palo Alto, CA, USA) was introduced and is now used for diverse clinical indications [8,9,10,11]. HyperArc™ provides several clinical and physical advantages over conventional radiotherapy techniques. Its design centers on optimized non-coplanar volumetric modulated arc therapy (VMAT) trajectories, which enable steep dose gradients and high conformality for intracranial targets. This dedicated platform utilizes an advanced optimization algorithm that automates the selection of non-coplanar couch and gantry angles, effectively avoiding collisions while maximizing the spatial separation of intersecting beams [12,13,14]. Recent studies have demonstrated that automated optimization of non-coplanar beam arrangements improves planning efficiency, reduces unnecessary couch rotations, and maintains high-quality dose distributions in single-isocenter SRS for multiple brain metastases. This automated angular mapping bypasses the historical limitations of manually selected trajectories, which were often constrained by institutional trial-and-error paradigms and conservative clearance margins. The platform’s ability to safely synthesize multiple overlapping arcs creates a unique spatial distribution of entry pathways, ensuring that no single normal tissue pathway receives excessive entry dose while establishing a tight geometric envelope around each lesion. This geometry maximizes dose fall-off, substantially reducing the integral dose to surrounding normal brain tissue.

By minimizing low-to-moderate dose spillage into healthy brain structures, HyperArc™ significantly lowers the risk of treatment-induced clinical outcomes [15,16,17]. Furthermore, the preservation of adjacent critical structures and healthy brain tissue becomes highly reproducible even when targets are numerous and closely clustered. Consequently, this high-gradient shielding capability directly supports long-term survivorship quality by mitigating the chronic decline in memory and executive function that traditionally accompanied large-volume cranial irradiation.

Furthermore, HyperArc™ streamlines clinical workflow through a single-isocenter approach for multiple lesions and automated delivery that eliminates manual couch adjustments. Together, these features enhance the precision of SRS and improve operational efficiency by reducing planning complexity and treatment delivery time [4,6].

Consequently, patient-specific quality assurance to verify such complex treatment plans has become increasingly critical. Because SIMT plans often involve highly modulated subfields, steep dose gradients, and off-axis target localization, even minor mechanical or dosimetric deviations can result in significant target underdosage or unexpected toxicities in normal tissues [18,19,20]. In these highly optimized delivery sequences, a microscopic deviation in leaf position or an uncorrected rotational setup error can shift the high-dose peak completely outside the target border of a small lesion, resulting in a severe localized geographical miss while simultaneously depositing uncompensated hot spots into healthy surrounding tissue. Conventional dosimetric tools, including ionization chambers, radiographic films, 2D arrays, and electronic portal imaging devices (EPIDs), have been used [2]. While traditional water-phantom or diode-matrix setups provide robust point-dose or planar measurements, they often suffer from limited spatial resolution or require labor-intensive alignment and additional phantom setup times [21,22,23,24]. The rigidity and bulk of traditional phantoms introduce operational bottlenecks, restricting a clinic’s ability to perform comprehensive daily pre-treatment verifications within a fast-paced schedule. Additionally, the discrete spacing between sensors in commercial diode matrices often fails to capture the intricate dose peaks and valleys characteristic of small-field stereotactic distributions.

Notably, EPIDs are increasingly preferred clinically because they allow efficient verification without additional phantom setups [25]. Furthermore, their modern amorphous silicon (a-Si) sensor arrays deliver excellent digital resolution with fine pixel pitches, making them exceptionally well-suited for capturing steep dose gradients in small stereotactic fields [26]. This transition to digital imagery enables medical physicists to evaluate delivery integrity in real time, capturing both the mechanical synchronization of the linear accelerator and the compound leaf positioning performance across multiple intersecting arcs. Consequently, the high data density provided by these advanced digital arrays opens new avenues for detecting localized multi-field irregularities that older dosimetric technologies routinely fail to resolve.

Despite the advantages of HyperArc™ in SIMT radiotherapy, Lalonde et al. [27] reported limitations in dose verification using EPIDs. They attributed these limitations to transmission and dosimetric leaf gap (DLG) values typically being derived as fixed parameters measured on the central axis (on-axis) using an ionization chamber, such as FC-65G.

However, under complex delivery conditions such as SIMT, factors including beam energy spectrum variations (e.g., beam hardening) and off-axis effects substantially increase uncertainty in portal dosimetry results. When multiple lesions are scattered far from the central axis, the radiation beam penetrates the peripheral region of the MLC leaves at oblique angles. Standard central-axis measurements using an ionization chamber do not accurately capture the spatial variations in transmission and effective leaf-end geometry that occur across wide-field, off-axis delivery profiles [28,29,30]. As the primary photon beam traverses the rounded boundaries of the off-axis leaves, the changing physical cross-section alters the penumbral slope and the transmitted energy spectrum. Because standard planning models project a uniform on-axis calibration baseline across the entire imager plane, they inherently miscalculate the cumulative leakage accumulated across large distances from the central ray.

Lalonde et al. [27] reported that the incorporation of energy-dependent corrections for transmission and DLG using EPIDs could improve the accuracy of portal dosimetry. However, the application of these corrections universally across clinical cases requires the careful consideration of operational efficiency [26,31]. Furthermore, commissioning and managing additional specialized prediction models within the treatment planning system introduces operational complexity, warranting clear institutional guidelines. Mitigating these systemic calculation anomalies is crucial because untreated mathematical offsets in secondary dose checks can mimic true machine delivery errors, leading to false-positive quality assurance (QA) failures.

This study therefore aimed to investigate the clinical impact of such parameter corrections by analyzing clinical data and comparing ionization chamber-based and EPID-based measurements as the target number increased. The objective was to evaluate variations in gamma passing rates with increasing target numbers in SIMT and to present an exploratory institutional observation and recommended internal action level for applying EPID-based corrections to ensure optimal portal-dose prediction agreement.

## 2. Materials and Methods

### 2.1. Ethics Statement

This retrospective study was approved by the Institutional Review Board (IRB) of Yeungnam University Medical Center (YUMC 2026-04-045). The requirement for informed consent was waived because patient anonymity was preserved.

### 2.2. Patient Selection

**Figure 1:**
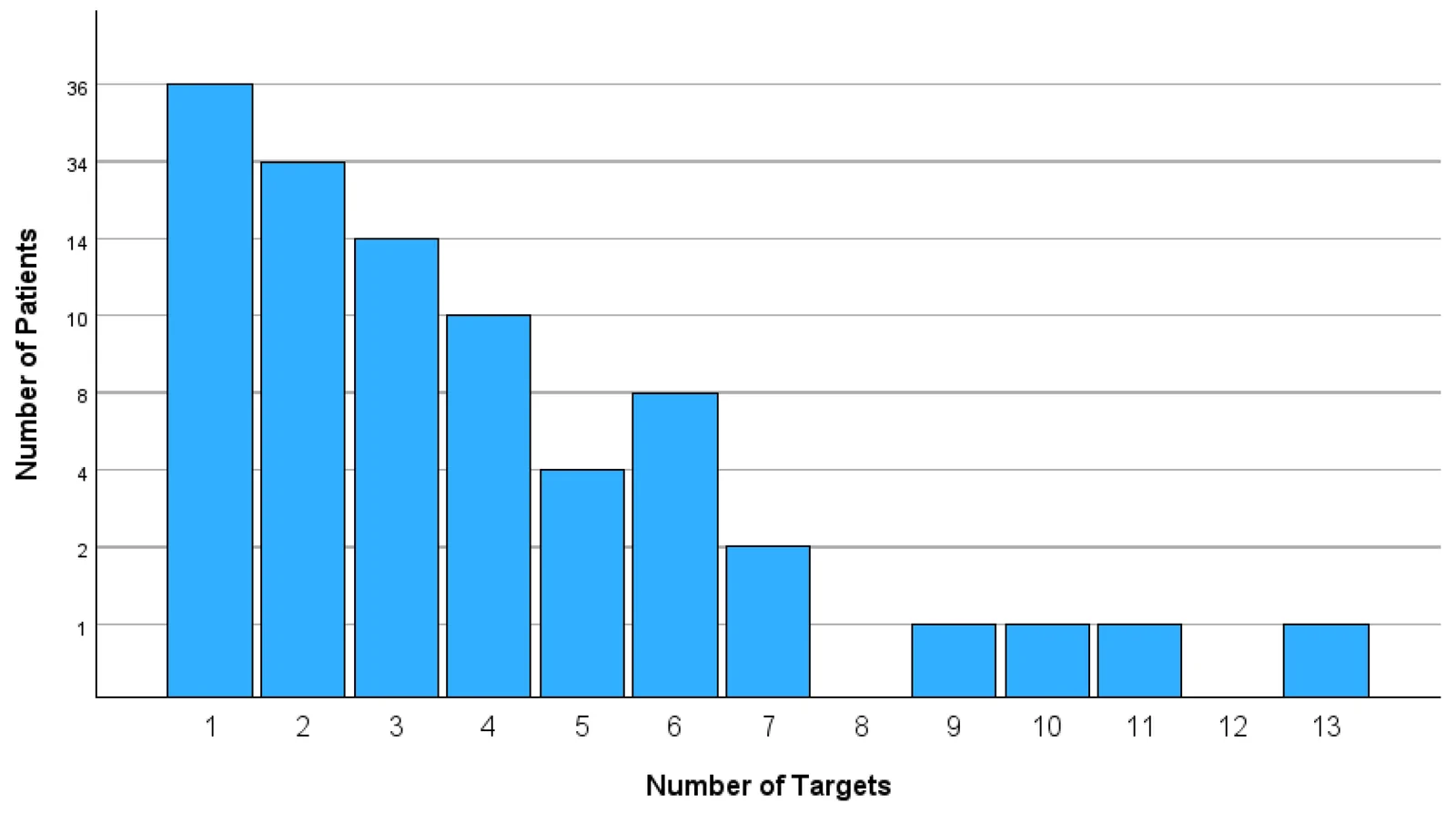
Distribution of targets per patient. The bar chart shows the frequency of patients according to the total number of intracranial targets planned for HyperArc-based stereotactic radiosurgery (SRS). In total, 112 patients were included, with targets ranging from 1 to 13.

The study included 112 consecutive patients who underwent HyperArc-based SRS between November 2022 and July 2025. Figure 1 shows the distribution of targets per patient, with the number of targets ranging from 1 to 13.

### 2.3. Radiation Treatment Planning

Patient immobilization, computed tomography simulation, and target delineation for HyperArc-based SRS were performed according to our institutional protocol, as previously described [5]. HyperArc beam geometry automatically configures four arcs: one at a couch angle of 0° and three at 45°, 90° (or 270°), and 315°. Most cases used the full four-arc configuration; however, in selected cases, a three-arc configuration was chosen based on tumor location.

All treatment plans were calculated using the Acuros XB algorithm (v15.6) for 6 MV Flattening Filter-Free (6X-FFF) beams from a Varian TrueBeam STx linear accelerator (Varian Medical Systems, Palo Alto, CA, USA) in the Eclipse treatment planning system. Photon-beam optimization used the Aperture Shape Controller set to “High,” the Dose Calculation Resolution was set to “Normal,” whereas the resolution for SRS or HyperArc™ was set to “High.”

All portal dosimetry plans were computed with the Portal Dose Image Prediction (PDIP) model (v15.6). Measurements were acquired using the AS1200 EPID (Varian Medical Systems, Palo Alto, CA, USA) mounted on the TrueBeam STx system. For portal dosimetry verification using the PDIP framework, all non-coplanar HyperArc fields were calculated and delivered in a converted QA geometry; the couch angle was set to 0° to avoid gantry-detector collision while maintaining identical MLC field sequencing, MUs, and beam modulation.

### 2.4. Transmission and DLG Measured by Ion Chamber

High-definition multileaf collimator (HD120 MLC, Varian Medical Systems, Palo Alto, CA, USA) transmission and DLG were measured using a water phantom. An FC65-G ionization chamber was positioned at a depth of 5 cm with a source-to-surface distance of 100 cm. MLC transmission (T) was evaluated using dedicated irradiation plans defined by the standard DICOM RT plan files provided by Varian Medical Systems. Measurements were acquired for fully open fields (10 × 10 cm^2^) and fields where either Bank A or Bank B leaves fully blocked the central beam axis. Transmission was calculated separately for each leaf bank using Equation (1):

(1) $$T_{\mathrm{bank}}=\frac{M_{\mathrm{blocked},\;\mathrm{bank}}}{M_{\mathrm{open}}}$$

where $M_{\mathrm{blocked},\;\mathrm{bank}}$ is the ionization reading with Bank A or Bank B closed, and $M_{\mathrm{open}}$ is the reading for the open field. The overall MLC transmission ($T_{\mathrm{IC}}$) was defined as the average transmission of Bank A and Bank B. To determine DLG, sliding gap widths of 2, 4, 6, 10, 14, 16, and 20 mm were used with the DICOM RT plan files provided by Varian Medical Systems. All measurements were conducted in triplicate (*n* = 3). The nominal gap width (g) was plotted on the vertical axis against the corrected detector reading ($R_{\mathrm{corr}}$) on the horizontal axis. Linear regression was performed according to Equation (2):

(2) $$g=a^{\prime }\cdot R_{\mathrm{corr}}+b^{\prime }$$

where $a^{\prime }$ represents the slope and $b^{\prime }$ denotes the vertical intercept (Y-intercept, $b^{\prime }<0$). The DLG value was directly derived as the absolute magnitude of the negative Y-intercept (i.e., $\mathrm{DLG}=|b^{\prime }|=-b^{\prime }$), representing the effective gap offset at zero reading.

### 2.5. Transmission and DLG Measured by EPID

Lalonde et al. suggested that, for SIMT, correcting transmission and DLG values using an EPID is necessary to improve PDIP calculation accuracy [27,32]. Instead of ion chamber measurements, corrected transmission and DLG values were derived using the averaged EPID reading within a 5 mm × 5 mm area at the central axis of each field. The same DICOM RT plan files and sliding gap widths provided by Varian Medical Systems were used for these measurements. To implement the EPID-derived corrections safely, a duplicate beam model was created in Eclipse Beam Configuration with the adjusted MLC transmission and DLG parameters assigned exclusively to its PDIP calculation properties. Portal-dose predictions were generated by copying the original clinical plan to this duplicate model, preserving identical monitor units and control-point leaf positions without altering the primary Acuros XB algorithm used for patient dose calculations. Measurements were performed using the identical set of DICOM RT plan files (open field, fully blocked Bank A/B fields, and seven sliding gap fields) and conducted in triplicate (*n* = 3). EPID-based transmission ($T_{\mathrm{EPID}}$) and DLG (${\mathrm{DLG}}_{\mathrm{EPID}}$) were evaluated using Equations (1) and (2) as described in Section 2.4.

### 2.6. Evaluation of Agreement Between Predicted and Measured Portal-Dose Images

Gamma index analysis [33] was performed using the Varian Portal Dosimetry software (v15.6) in absolute dose mode without the manual factor modification. Portal-dose agreement was evaluated using the global gamma normalization method relative to the maximum predicted dose (Calibration Units, CU), with the “Use Local Gamma Evaluation” option disabled. The spatial alignment of the measured image relative to the predicted dose was performed using the automated alignment tool (Auto Align using Reference Dose) restricted strictly to translational shifts along the X and Y axes, while rotational alignment was maintained at zero degrees ($0.00^{\circ }$). To exclude background radiation and pixels outside the irradiated area, analysis was restricted to the irradiated field area (Field + ROI) with a 10% low-dose threshold.

To maintain direct comparability with the baseline methodology of Lalonde et al. [27], portal-dose agreement was evaluated using the 3%/2 mm criterion with a 95% pass rate threshold, as recommended by the American Association of Physicists in Medicine (AAPM) Task Group 218 report [22]. Detector calibration was performed during routine monthly quality assurance according to standard Varian protocols (including dark field, flood field, and absolute dose CU calibration).

### 2.7. Statistical Analysis

Continuous variables were expressed as mean ± standard deviation as well as median and interquartile range (IQR) to account for data skewness near upper boundaries. Normality of paired differences was evaluated using the Shapiro–Wilk test. Differences in gamma passing rates between uncorrected and EPID-corrected methods were assessed using both the paired t-test and the non-parametric Wilcoxon signed-rank test. Pass/fail status across methods based on the 95% threshold was evaluated using McNemar’s test. Spearman’s rank correlation coefficients (*r_s_*) with 95% confidence intervals (CI) were calculated. A p-value < 0.05 was considered statistically significant. Statistical analyses were performed using SPSS version 29.0 (IBM Corp., Armonk, NY, USA).

## 3. Results

### 3.1. Transmission and DLG Values

**Figure 2:**
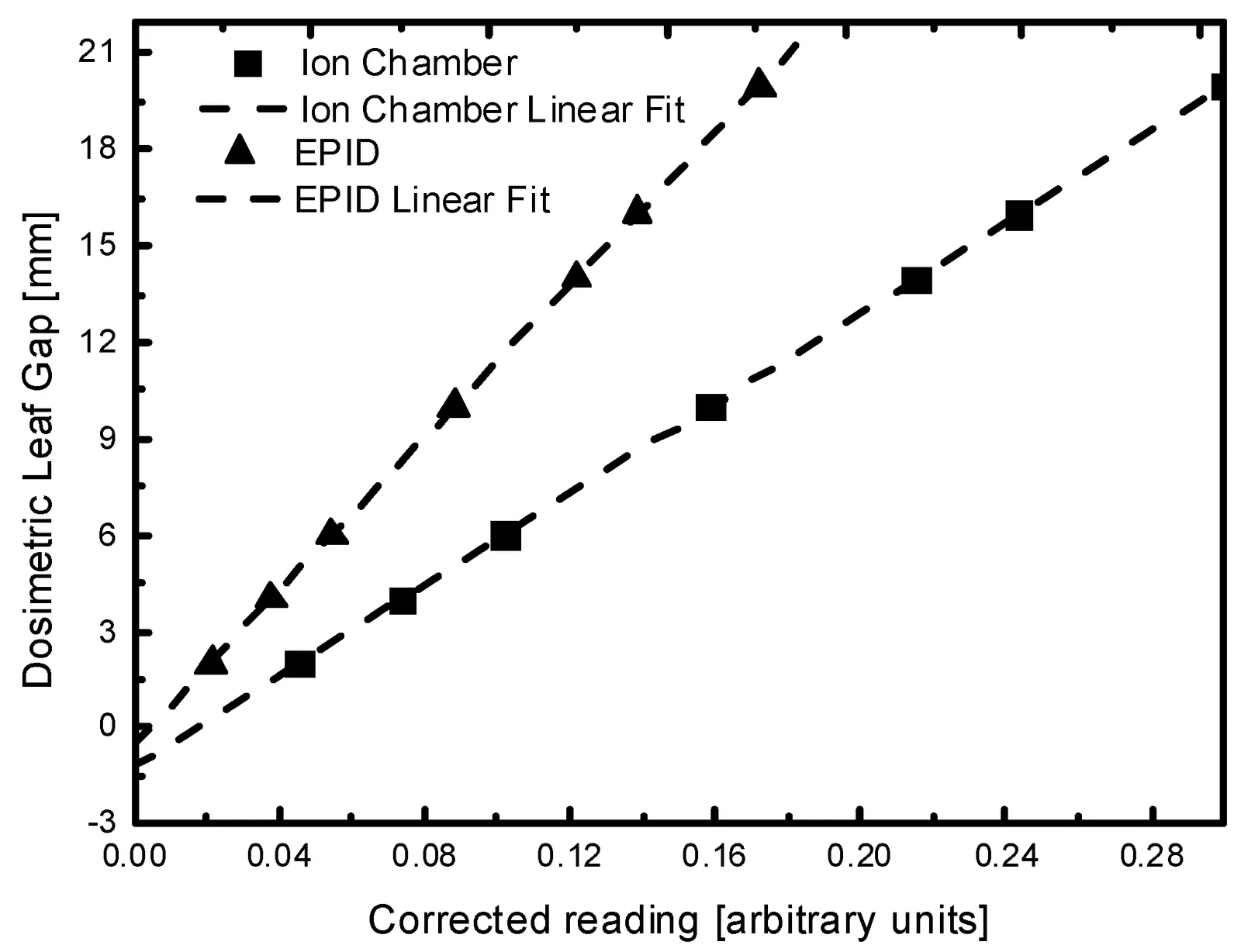
Comparison of dosimetric leaf gap (DLG) determination using an ion chamber (squares) and EPID (triangles). The linear regression plots display the nominal leaf gap width (Y-axis, mm) against corrected detector readings (X-axis, arbitrary units). High linearity was observed for both detectors (*R*^2^ = 0.9999). The DLG values (0.1210 mm for the ion chamber and 0.0500 mm for the EPID) were derived as the absolute magnitude of the negative Y-intercepts ($\left|b^{\prime }\right|$) obtained from the linear regression model ($g=a^{\prime }\cdot R_{\mathrm{corr}}+b^{\prime }$). DLG, dosimetric leaf gap; EPID, electronic portal imaging device.

MLC transmission and DLG values were measured in triplicate (*n* = 3) using both the FC65-G ionization chamber and AS1200 EPID. For the ion chamber, the average MLC transmission was 0.0078. For the EPID, the corresponding average transmission was 0.0066. Figure 2 presents the linear regression analysis for DLG determination, plotting nominal gap widths (Y-axis, mm) against corrected detector readings (X-axis, arbitrary units). Linear regression analysis demonstrated excellent fit for both detectors (*R*^2^ = 0.9999 for EPID and *R*^2^ = 0.9999 for ion chamber). From the fitted regression model, the derived DLG values—calculated directly as the absolute magnitude of the negative Y-intercepts ($\left|b^{\prime }\right|$)—were 0.1210 mm for the ion chamber and 0.0500 mm for the EPID. These adjusted parameters, obtained using the methodology proposed by Lalonde et al., were used to optimize the PDIP model.

### 3.2. Qualitative Comparison of Portal-Dose Distributions

**Figure 3:**
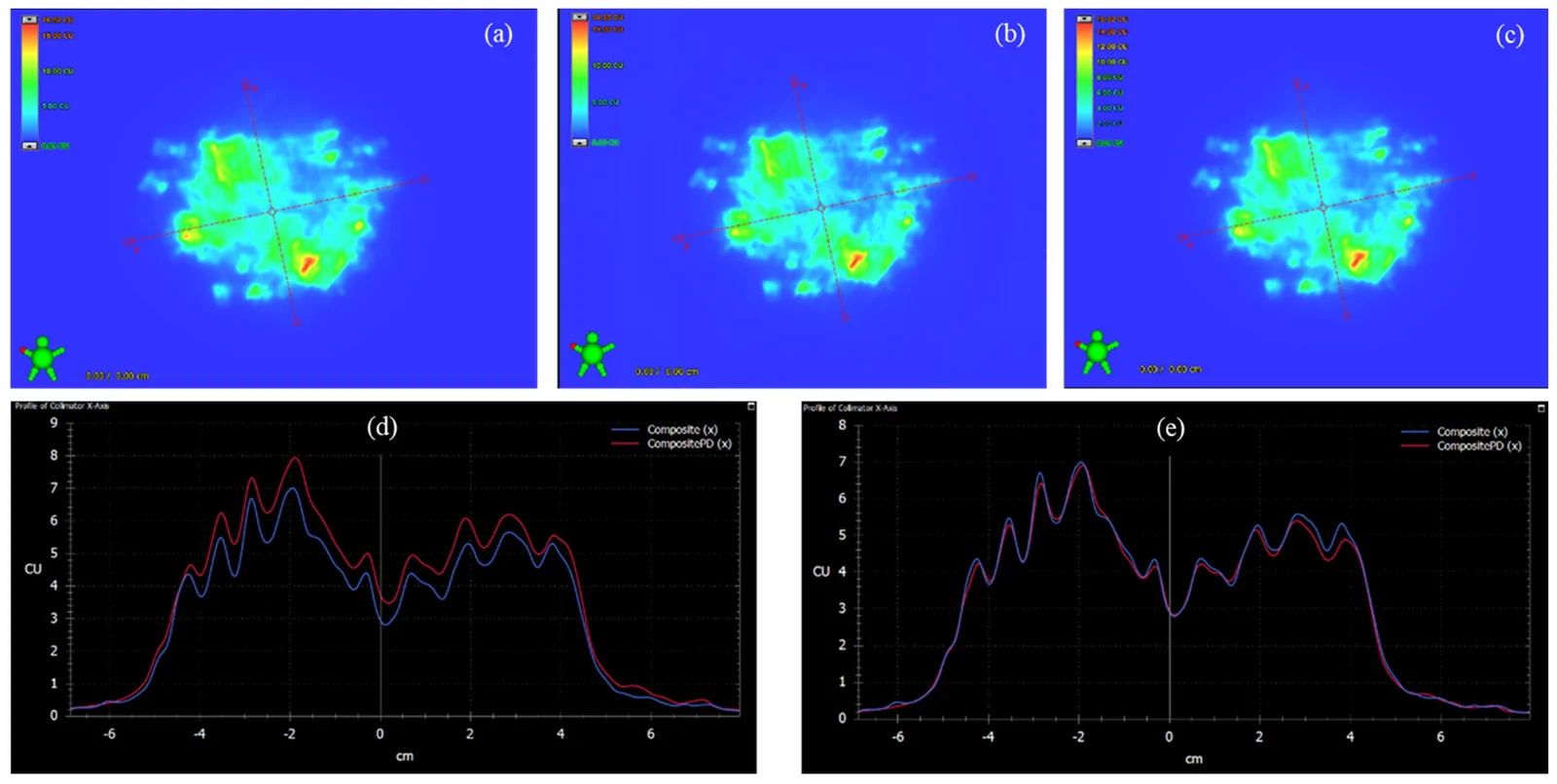
Composite portal-dose image of HyperArc radiation therapy treating nine targets simultaneously with a single isocenter. (**a**) Predicted portal dose using the uncorrected method (transmission and DLG measured using an ion chamber); (**b**) portal dose measured using EPID; (**c**) predicted portal dose measured using the Lalonde-corrected method (transmission and DLG measured by EPID); (**d**) measured (blue line) versus predicted (red line) portal-dose profile in Calibration Units (CU) across the central *X*-axis (*Y* = 0 cm) of the composite portal-dose distribution for the nine-target plan without the EPID-based multileaf collimator (MLC) transmission and DLG correction; (**e**) measured (blue line) versus predicted (red line) portal-dose profile in Calibration Units (CU) across the central *X*-axis (*Y* = 0 cm) of the composite portal-dose distribution for the nine-target plan with EPID-based MLC transmission and DLG correction. EPID, electronic portal imaging device; DLG, dosimetric leaf gap; CU, calibration unit.

The impact of EPID-based MLC transmission and DLG correction was qualitatively evaluated using a representative nine-target SIMT case (Figure 3). With the uncorrected method (transmission and DLG measured by an ion chamber), the predicted portal-dose image (Figure 3a) showed visible discrepancies compared with the measured EPID image (Figure 3b). This was further supported by dose profile analysis (Figure 3d), in which the uncorrected predicted dose (red line) consistently overestimated the measured dose (blue line). In contrast, after applying Lalonde et al.’s EPID-based correction, the predicted portal-dose distribution (Figure 3c) and corresponding profile (Figure 3e) showed excellent agreement with the measured data, mitigating the overestimation observed in the uncorrected model.

### 3.3. Overall Gamma-Analysis Performance

**Figure 4:**
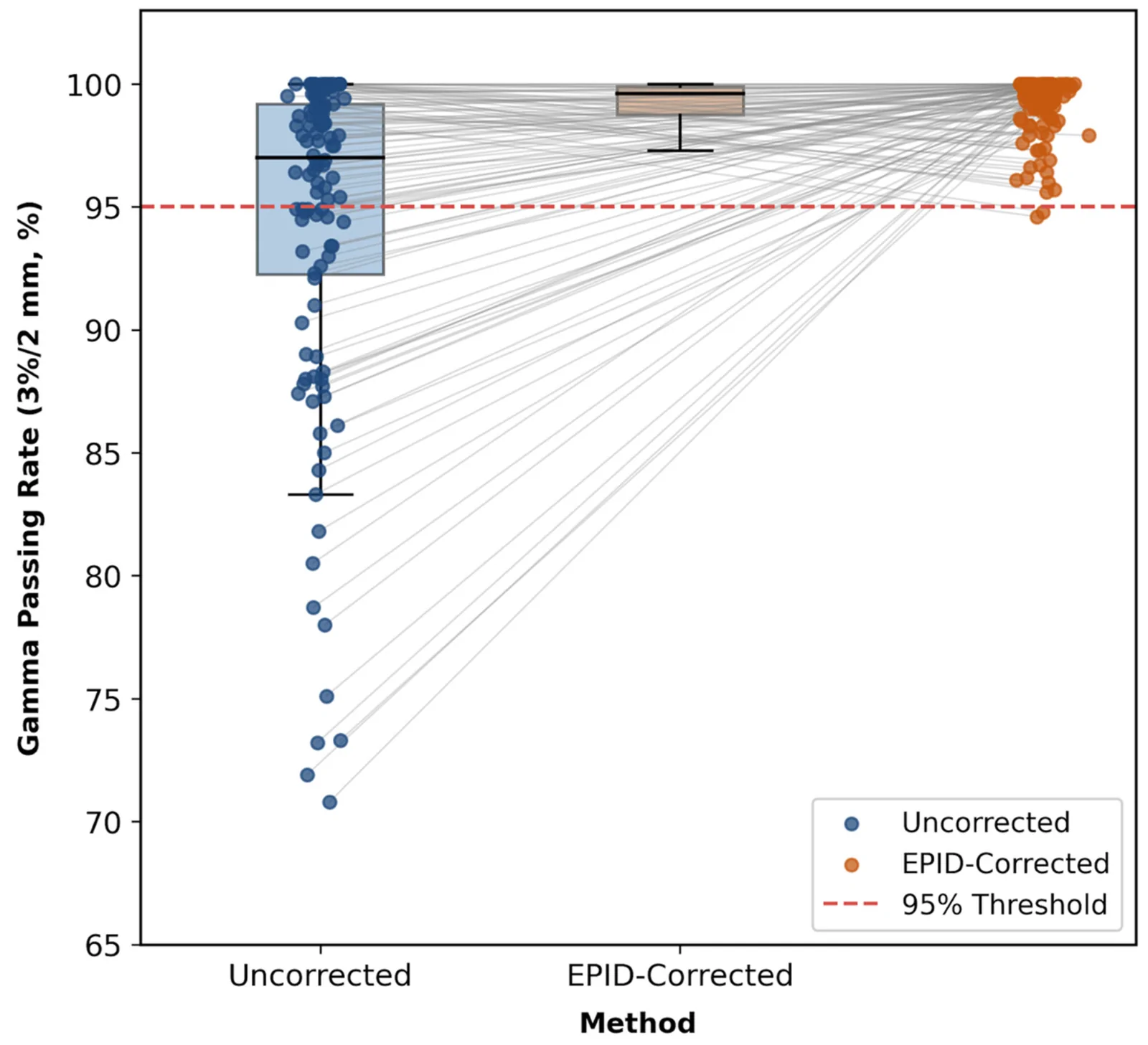
Paired comparison of gamma passing rates (3%/2 mm) between the uncorrected method and Lalonde et al.’s EPID-based correction method across all 112 patient plans. Individual paired measurements are connected by gray lines, overlaid with boxplot distributions displaying the median, interquartile range (IQR), and overall data spread. The red dashed line denotes the 95% clinical tolerance threshold. Paired analysis demonstrated a statistically significant improvement post-correction (p<0.001).

The quantitative accuracy of portal-dose prediction was assessed using gamma index analysis (3%/2 mm) across all 112 analyzed plans (Figure 4). The uncorrected method demonstrated substantial variation in gamma passing rates (mean: 94.28 ± 7.03%; 94.28 ± 7.03%; median: 97.00%, IQR: 92.25–99.20%), with 43 of 112 plans (38.4%) failing to meet the 95% clinical tolerance threshold (lowest rate: 70.8%). In contrast, Lalonde et al.’s correction method significantly improved passing rates (mean: 99.07 ± 1.25%; median: 99.60%, IQR: 98.75–99.90%), reducing failures to only 2 out of 112 plans (1.8%).

The Shapiro–Wilk test indicated non-normality of paired differences (*p* < 0.001), justifying the use of non-parametric testing. Both the paired *t*-test (t_111_ = 6.84; *p* < 0.001) and Wilcoxon signed-rank test (W = 889.0, *p* < 0.001) confirmed a statistically significant improvement post-correction. McNemar’s test further demonstrated a significant reduction in plan failure proportion (*p* < 0.001).

### 3.4. Correlation Between Target Count and Portal-Dose Prediction Accuracy

**Figure 5:**
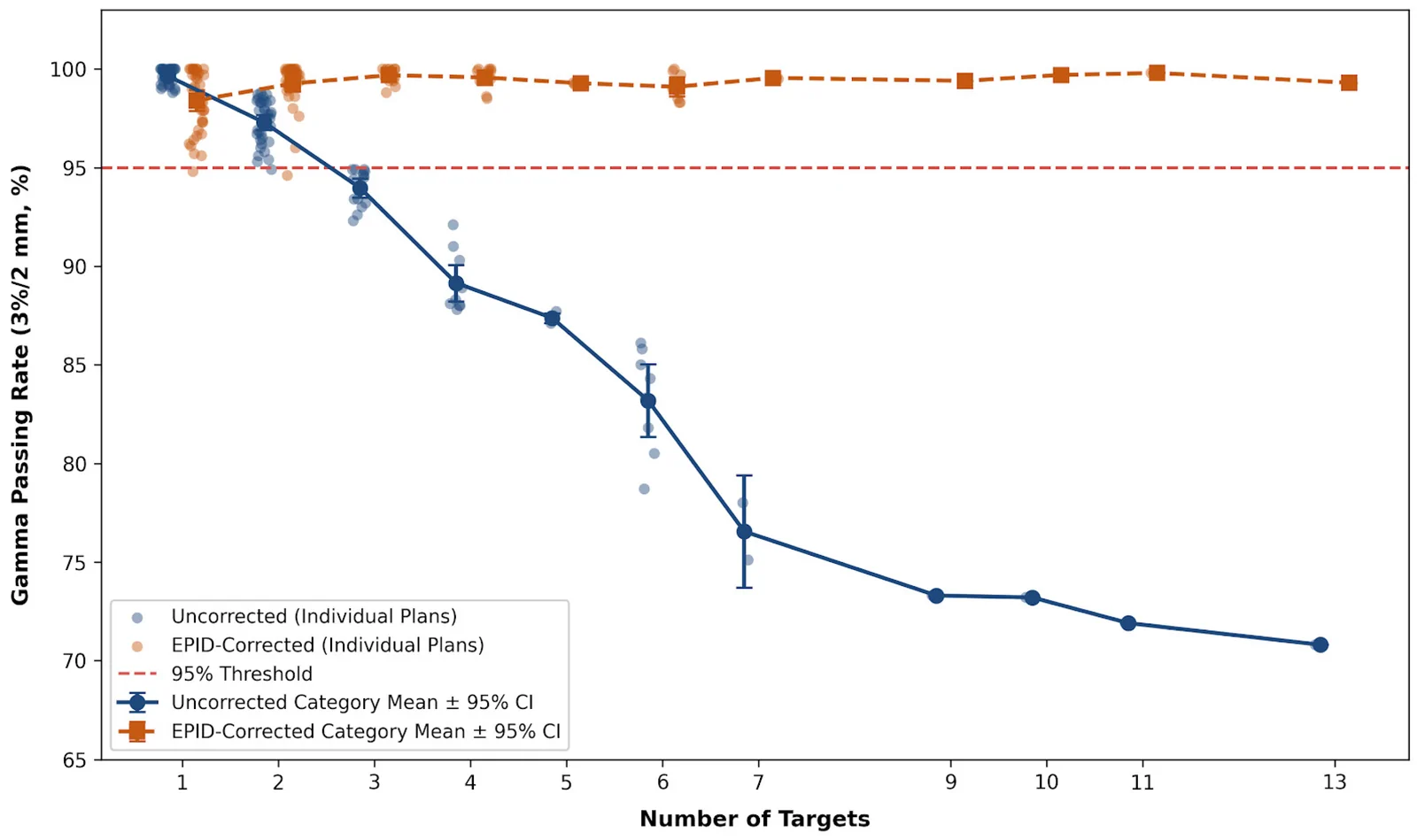
Plan-level gamma passing rates (3%/2 mm) and category distributions as a function of target count. Individual plan observations are displayed as jittered scatter points (blue circles for uncorrected; orange squares for EPID-corrected). The category mean values are overlaid with 95% confidence interval (CI) error bars. The uncorrected method demonstrated a strong negative Spearman rank correlation (r_s_ = −0.773, *p* = 0.005), declining sharply with increasing target complexity; the EPID-corrected method maintained consistently high passing rates regardless of target count (r_s_ = 0.118, *p* = 0.729). The red dashed line denotes the 95% clinical tolerance threshold. Sample sizes (*n*) per target count category are as follows: 1 target (*n* = 36), 2 targets (*n* = 34), 3 targets (*n* = 14), 4 targets (*n* = 10), 5 targets (*n* = 4), 6 targets (*n* = 8), 7 targets (*n* = 2), and 9, 10, 11, and 13 targets (*n* = 1 each).

The relationship between intracranial target number and portal-dose prediction accuracy was further analyzed using Spearman’s rank correlation (Figure 5). With the uncorrected method, gamma passing rates declined sharply as the target number increased, showing a statistically significant and strong negative correlation (r_s_ = −0.773, 95% CI: −0.949 to −0.234, *p* = 0.005). This observation indicates that cumulative MLC transmission and DLG errors become progressively pronounced in complex SIMT plans. However, after applying Lalonde et al.’s correction, gamma passing rates remained stable and consistently high (>98%), showing no significant correlation with target count (r_s_ = 0.118, 95% CI: −0.520 to 0.672, *p* = 0.729).

For plans with one or two targets, the uncorrected method still yielded gamma passing rates within the clinical tolerance threshold. However, when complexity reached three or more targets, passing rates declined markedly. This finding indicates that EPID-based correction effectively compensates for MLC-related dosimetric uncertainties, even in highly complex SRS cases with multiple lesions. Therefore, while conventional ion chamber-based values may suffice for simple one-target or two-target plans, the three-target level serves as a descriptive institutional observation, indicating where EPID-based parameter calibration may provide beneficial prediction agreement in multi-target SRS workflows.

## 4. Discussion

Accurate verification of SIMT plans remains a major challenge in modern radiation oncology because of the high complexity of multi-target delivery and the sensitivity of stereotactic dose gradients. This study evaluated the EPID-based MLC transmission and DLG correction method proposed by Lalonde et al. [21,27] and, for the first time, established a practical exploratory target count-based institutional observation. Identifying such a baseline is highly valuable for busy clinical departments where balancing absolute dosimetric accuracy with time efficiency is essential. As the prevalence of multi-lesion radiosurgery grows, optimizing patient-specific QA protocols represents a crucial step toward standardization.

Our results showed clear differences between parameters measured using a conventional ion chamber and those derived from EPID. DLG decreased from 0.1210 mm (ion chamber) to 0.0500 mm (EPID), while the transmission factor decreased from 0.0078 to 0.0066. This discrepancy occurs because ion chamber measurements are typically performed on-axis in a water phantom, whereas the PDIP model depends on the EPID-specific response to the photon spectrum. Because SIMT plans involve many small off-axis fields, standard on-axis ion chamber parameters do not account for variations in beam quality and leaf-end effects across the wider detector area. The lower DLG and transmission values obtained using EPID mitigated the dose overestimation observed in the uncorrected profiles (Figure 3), aligning the predicted fluence more closely with the measured physical delivery. An amorphous silicon (a-Si) EPID panel exhibits distinct cross-sectional sensitivity and energy response variations compared to gas-filled ionization chambers. When exposed to unflattened (FFF) beams, off-axis softening of the energy spectrum significantly alters how leaf-end transmission and penumbra structures are recorded, explaining why optimization using direct EPID measurements yields a more accurate prediction baseline within the PDIP framework [28,31,34]. This mismatch highlights that a water-equivalent calibration environment cannot intrinsically simulate the solid-state semiconductor interactions occurring during peripheral field tracking. Because the PDIP algorithm projects raw pixel metrics into a predicted dose distribution, using parameters derived from non-native detector geometry introduces systematic artifacts that worsen under intense off-axis scatter conditions.

This study showed a strong negative correlation between target number and the mean gamma passing rate in uncorrected plans (r_s_ = −0.773, 95% CI: −0.949 to −0.234, *p* = 0.005; Figure 5). In SIMT delivery, the MLC leaves spend considerable time in the “closed” or “sliding” positions to shield areas between multiple lesions. This produces cumulative modeling discrepancies; small overestimations of transmission and DLG for each individual target-field segment accumulate, resulting in a substantial total dose discrepancy. This explains why plans with nine or 13 targets had gamma passing rates as low as 70.8%, which were well below the 95% clinical threshold recommended by AAPM TG-218 [22].

As target count increases, the total number of monitor units (MUs) and control points generally scale upward. This higher modulation increases the fractional contribution of leaf-transmission dose relative to open-field dose. Consequently, even minute overestimations in baseline MLC leakage parameters, compounded across dozens of highly modulated field segments, progressively drive down gamma passing rates in a linear fashion [27]. These systematic errors act like artificial background noise, inflating valleys between adjacent high-dose peaks. When multiple small lesions are tightly clustered, this artificial background buildup fills in steep dose gradients, causing severe localized discrepancies where the imager expects complete radiation nulls.

While the EPID-derived model is commissioned once per machine, the management of dual prediction models within routine clinical workflows requires practical operational thresholds. Our analysis suggests that, for simpler plans involving one or two targets, conventional ion chamber-based parameters may still provide acceptable passing rates within the 95% tolerance. This is likely because the cumulative MLC error is not yet large enough to exceed the gamma criteria. In these low-complexity setups, the target volumes are frequently positioned closer to the central axis, meaning that the spatial impact of off-axis beam variations remains minimal and well within the native tolerances of standard calculation models [22]. The absolute volume of shielding leaves remaining closed is comparatively restricted in these simple configurations, preventing the baseline modeling errors from overwhelming the primary core fluence calculations.

However, we observed a marked decline in passing rates starting at three targets. Therefore, we present the use of three targets as an exploratory institutional observation rather than an evidence-based clinical action level. Applying this method to plans with three or more lesions supports improved portal-dose prediction agreement while maintaining operational efficiency in simpler cases. This tailored stratification optimizes clinical workflow: medical physicists can bypass intensive parameterization for straightforward one- or two-target treatments and instead focus resources on optimizing QA for plans with three or more targets, where dosimetric deviation poses a genuine risk. This clear stratification offers a practical institutional reference to streamline QA workflows. It eliminates the operational inertia of treating all cases with a single monolithic approach, allowing resources to be deployed precisely where complexity warrants additional validation.

Our findings support those of Lalonde et al. [27], who emphasized that standard DLG values are insufficient for complex SIMT SRS. Although their work established the methodology, this study extends the evidence by quantifying the relationship between target count and passing rates in a cohort of 112 patients. The consistent mean passing rate of 99.07 ± 1.25% achieved post-correction (r_s_ = 0.118, *p* = 0.729; Figure 5) indicates that this calibration method effectively reduces model calculation discrepancies for HyperArc-based treatments under our institutional configuration. However, this improvement reflects the enhanced agreement between EPID measurements and the EPID-specific PDIP calculation model; it does not independently demonstrate improved absolute patient-dose delivery or establish a universally applicable clinical cutoff.

This study has several limitations. First, it was restricted to 6X-FFF beams, which limits its generalizability. Although this is the most commonly used energy for SRS, other beam energies may behave differently; thus, further investigation is needed to determine whether the proposed exploratory action level applies to other energies or MLC types. Second, the analysis was performed on a single platform using the HD120 MLC, whose fine 2.5 mm central leaves and distinct tongue-and-groove layouts and cross-leaf leakage patterns may not reflect performance in other configurations—such as the Millennium 120 MLC with 5.0 mm leaves; this may shift the precise onset of significant cumulative modeling discrepancies. Future multicenter validations encompassing various linear accelerator setups and energy combinations will be essential to confirm broader applicability. Third, a fundamental limitation of this study is that corrected MLC parameters were derived from EPID measurements and were subsequently evaluated against predicted portal-dose images using the same EPID detection system. While this evaluation demonstrates that EPID-derived parameters effectively resolve modeling discrepancies within the PDIP framework for complex off-axis fields, it does not constitute an independent validation of the physical dose delivered to the patient, nor does it establish a universal workflow threshold. Thus, future studies incorporating independent dosimetric toolsets—such as high-resolution film, gel dosimetry, or 3D diode arrays in anthropomorphic phantoms—are required to verify actual delivered patient dose and absolute delivery accuracy. Furthermore, it is important to emphasize that potential confounding factors—such as total monitor units, target-to-isocenter distance, individual target volumes, aperture characteristics, number of control points, and specific arc configurations—were not systematically evaluated in relation to gamma passing rates. Additionally, no formal statistical breakpoint analysis, ROC evaluation, or bootstrap sensitivity testing was performed to establish an absolute clinical cutoff. Consequently, the proposed three-target threshold represents a purely descriptive institutional observation based on our single-center experience within the PDIP framework, rather than an evidence-based clinical action level or standardized guideline.

## 5. Conclusions

In this retrospective study, the EPID-based correction of MLC transmission and DLG significantly improved portal-dose prediction agreement in HyperArc-based SIMT SRS. While conventional ion chamber-based parameters remained adequate for simple one-target or two-target plans, dose-prediction consistency declined markedly starting at three targets. Incorporating EPID-specific parameter calibration effectively mitigated cumulative off-axis modeling errors, yielding consistently high gamma passing rates regardless of plan complexity. Overall, we present the three-target threshold as a descriptive institutional observation, offering a practical reference to optimize portal QA efficiency in multi-target SRS workflows.

## Data Availability

The original contributions presented in this study are included in the article. Further inquiries can be directed to the corresponding author.

## Abbreviations

The following abbreviations are used in this manuscript:

SIMT	single-isocenter multiple targets
SRS	stereotactic radiosurgery
MLC	multileaf collimator
EPID	electronic portal imaging device
DLG	dosimetric leaf gap
VMAT	volumetric modulated arc therapy
PDIP	portal-dose image prediction
YUMC	Yeungnam University Medical Center
CT	computed tomography
FFF	flattening filter-free
QA	quality assurance
AAPM	American Association of Physicists in Medicine
TG-218	Task Group 218 (AAPM report)
SBRT	stereotactic body radiotherapy
MU	monitor units
a-Si	amorphous silicon
